# Contrasting Narratives of Race and Fatness in Covid-19

**DOI:** 10.1007/s40656-021-00477-5

**Published:** 2021-11-18

**Authors:** Azita Chellappoo

**Affiliations:** 1 Department of Philosophy I, Ruhr Universität Bochum, Universitätsstrasse 150, 44801 Bochum, Germany; 2 Present Address: Department of Philosophy, Faculty of Arts and Social Sciences, The Open University, Walton Hall, Milton Keynes, MK7 6AA UK

**Keywords:** Race, Covid-19, Fatness, Obesity, Health, Inequality

## Abstract

The slogan that ‘the virus doesn’t discriminate’ has been belied by the emergence of stark and persistent disparities in rates of infection, hospitalisation, and death from Covid-19 between various social groups. I focus on two groups that have been disproportionately affected, and that have been constructed or designated as particularly ‘at-risk’ during the Covid-19 pandemic: racial or ethnic minorities and fat people. I trace the range of narratives that have arisen in the context of explaining these disparities, in both the scientific literature and wider expert and public discourse. I show that the scientific and public narratives around these groups have differed significantly, revealing contested and competing conceptions of the basis of these categories themselves. These different conceptions have important impacts on the kinds of interventions that become possible or desirable. I show that in the case of racial or ethnic disparities, genetic narratives have been combatted by a strong focus on structural racism as a driver of pandemic inequalities. However, in the case of fatness, individualising and stigmatising narratives have dominated discussions. I suggest that, given racial or ethnic differences in prevalence of fatness, and scholarship casting anti-fatness as historically racialised, the stigmatisation of fatness disproportionately affects racial or ethnic minorities in terms of placing individual blame or responsibility for the increased burden of Covid-19 on these groups. Despite widespread acknowledgement of the role of structural racism in driving racial inequalities in the burden of Covid-19, anti-obesity rhetoric and research provides a ‘backdoor’ to placing blame on individuals from racial minorities.

## Introduction

The emergence of social disparities in the burden of Covid-19 has been a defining feature of the pandemic (Abedi et al., 2021; Islam et al., 2021; Stok et al., 2021). This has opened up conversations around disparate health outcomes, social inequality, and structural discrimination (Siddique, 2020; Yearby, 2021). In this paper I focus on two broad groups that have been constructed or designated as particularly ‘at-risk’ during the Covid-19 pandemic: racial or ethnic minorities and fat people.^1^ As the disparities in Covid-19 related outcomes (including risk of infection, rates of hospitalisation and rates of mortality) have become evident, this has led to scientific research investigating possible explanations for these disparities, including genetic research (examples include Hou et al., 2020; McCoy et al., 2020) and epidemiological research (examples include Garcia et al., 2021; Ruprecht et al., 2021). This has not been confined to the academic literature: there has also been widespread expert and public discourse surrounding the causes and necessary remedies of disparities in the burden of Covid-19, including articles in popular media outlets such as The Guardian (Boseley, 2021; Morgan, 2020) and The New York Times (Kolata, 2020; Stolberg, 2020), as well as the dissemination of expert opinion in venues such as Scientific American (Gravlee, 2020) and the commentary sections of the Lancet (Bhala et al., 2020) and the British Medical Journal (Davies & Ghezzi, 2020; Palmer, 2020).

Both people of colour and fat people are salient social categories, which in some contexts have been understood as biological categories, and in both these cases there have been stark documented disparities in the effects of Covid-19. However, the scientific and public narratives around these groups have differed significantly, revealing contested and competing conceptions of the basis of these categories themselves.^2^ In a time of uncertainty and rapidly changing biomedical knowledge, these narratives have been taken up in diverging ways. This is important not just for a philosophical interrogation of our conceptions of race and fatness: the ways in which these categories have been understood in both the scientific literature and media reporting open up, or close off, particular kinds of social, political, or biomedical interventions.

In Sect. 2 I outline the evidence for racial and ethnic disparities in the burden of Covid-19, and then delineate three broad narratives that have structured our understanding of these disparities: genetic, sociostructural, and biosocial. I show how genetic narratives emerged within the scientific literature, although sociostructural narratives were swiftly advanced to combat explanations that rely on genetic concepts of race. Biosocial explanations that attend to the impact of forces such as structural racism on biological processes have also been advanced in the context of the apparent debate over biological or social causes, although this narrative has not received widespread attention. In Sect. 3 I outline the evidence for fat-thin disparities, and then delineate three broad narratives that have structured our understanding in this case: personal responsibility and the ‘second pandemic’, social and environmental drivers, and structural discrimination. I show that, while individualising and stigmatising narratives of innate inferiority have received forceful pushback in the case of racial or ethnic disparities, these models have dominated discussion of fatness in the pandemic. In Sect. 4 I show that these twin disparities should not be considered in isolation: given racial or ethnic differences in prevalence of fatness, and scholarship casting anti-fatness as historically racialised, the stigmatisation of fatness disproportionately affects racial or ethnic minorities.

## Racial and Ethnic Disparities in Incidence and Severity of Covid-19

As the number of Covid-19 cases rose across the globe, racial and ethnic disparities in the incidence and severity of Covid-19 have been increasingly documented. In the United States, Black people have died from Covid-19 at 1.9 times the rate of white non-Hispanic people, Hispanic or Latino people have died at 2.3 times the rate, and Indigenous people have died at 2.4 times the rate (as per data aggregated by the Centers for Disease Control and Prevention (CDC), April 2021b). These groups are also becoming infected with coronavirus and becoming hospitalised with Covid-19 at much higher rates.

Similar disparities have been seen elsewhere. In the United Kingdom, the first ten doctors that were known to have died from the virus were all BAME (Black, Asian, and minority ethnic) individuals. Statistics bore out the existence of racial or ethnic disparities: after correcting for age, Black men are 4.2 times more likely to die from Covid-19, and Black women are 4.3 times more likely, than white people in England and Wales (Office for National Statistics, May 2020a). People of Bangladeshi and Pakistani ethnicities are also significantly more likely to die from Covid-19 compared to white people (ibid). These disparities still persist after controlling for socioeconomic circumstances, demographic characteristics, and health or disability (for example, after taking these factors into account, Black people are still 1.9 times as likely, and Bangladeshi and Pakistani people are 1.8 times as likely, to die from Covid-19).

This has led to calls from scientific, public health, and medical experts, as well as from politicians, public figures and the public at large, for greater investigation into the causes of and remedies for the disproportionate impact of Covid-19 on racial or ethnic minorities (American Medical Association et al., 2021; Siddique, 2020; Warren et al., 2020). As these discourses have developed, conflicting and competing narratives have arisen regarding the causal pathways underlying these disparities. These narratives are rooted in pre-existing approaches to racial difference, as well as deeper commitments to particular race concepts. However, they have taken on novel forms, and prompted new reactions or kinds of uptake in the rapidly shifting information landscape that has characterised pandemic knowledge. Three causal explanations have been put forward for the racial or ethnic disparities in incidence and severity of Covid-19. These are, firstly, genetic differences between racial or ethnic groups (either as a key causal factor or as one causal factor among many), secondly, sociostructural differences that point to socially constructed racial hierarchies that drive systematic differences in the material conditions of racial or ethnic groups, and, lastly, biosocial approaches that indicate the role of interactions between social conditions and embodied difference (such as through epigenetic mechanisms). Whilst genetic and sociostructural narratives have received sustained attention in both scientific literature and popular media, biosocial approaches have been comparatively sidelined. In addition, the ways that each narrative frames racial or ethnic categories or groups can mask or highlight heterogeneity within these groups, and make legible particular kinds of political or social interventions.

### Genetic Narratives of Racial and Ethnic Disparities

As the racial and ethnic disparities emerging in the pandemic became clear, genetic explanations for these inequalities quickly began to surface. This generated scientific studies probing racial genetic factors, as well as news coverage and public debate. Bunyavanich et al (2020) report that nasal gene expression of transmembrane serine protease 2 (TMPRSS2) differs between racial groups. TMPRSS2 activates the SARS-CoV-2 spike protein and therefore facilitates virus entry to the body; the authors suggest that higher expression of TMPRSS2 in Black individuals contributes to the increased burden of Covid-19. Singh and Wurtele (2020) identify a subset of differentially expressed genes between African Americans and European Americans which are known to be implicated in infection, inflammation and immunity. It is important to note that gene expression is a function not only of DNA sequence, but also of environmental factors (through epigenetic processes), and the existence of differences in gene expression profiles in itself does not suggest racial genetic variation. The biosocial perspective on racial disparities in Covid-19 will be discussed in Sect. 2.3. Despite this qualification, these studies have been reported as searches for genetic factors (Robertson, 2020).

Other researchers have been more direct about the potential genetic role: McCoy et al (2020) point to racial genetic variations in the androgen receptor as a contributing causal factor, inferring this from studies implicating the androgen receptor in gender differences in Covid-19 disease severity and mortality, as well as previous work indicating there are racial variations in androgen-mediated conditions such as prostate cancer. Hou et al. (2020) suggest polymorphisms in the ACE-2 and TMPRSS2 genes as potential explanations for differential racial susceptibility. In a review article, Ovsyannikova et al (2020) state that it is “well known that individuals of diverse racial and ethnic backgrounds harbor different allelic variants” (210), and suggest genetic differences may explain racial differences in clinical outcomes observed in the US.

Some researchers have acknowledged the impact of social factors such as discrimination and psychosocial stress in generating health disparities, whilst still maintaining that genetic factors play a key role. Williamson et al. (2020) analysed the health records of 17 million NHS patients in the UK and found that Asian and Black individuals were at significantly increased risk of in-hospital death from Covid-19. These groups were still at increased risk even after accounting for age, sex, smoking status, a range of comorbidities (including asthma, diabetes, and chronic heart disease), and deprivation (measured by the Index of Multiple Deprivation, derived from postcodes). Whilst Williamson et al. (2020) do not speculate on causal relationships, these results were taken by Phillips et al. (2021) to indicate that “comorbidity and social determinants of health only tell part of the story” (4) when it comes to racial and ethnic disparities, and so, “*[n]aturally*, biological risk likely fills the void” (ibid, emphasis mine). The inability for particular chosen measures of risk to eliminate racial disparities is taken as evidence for their hypothesis that ‘ancestral variation’ in polymorphisms of genes involved in the immune response is a causal factor in the increased susceptibility of Black Americans to Covid-19.

In a response to the reporting of the Williamson et al. study in the British Medical Journal, Kevin Davies and Pietro Ghezzi, Professors of Medicine at Brighton and Sussex Medical School, also read a genetic narrative into the Williamson et al. data, suggesting that genetic variations in two immunoglobulin Fc-receptors “could potentially explain the more severe disease observed in BAME patients”, and urging scientists to focus on investigating these genes “as a priority” (Davies & Ghezzi, 2020). Others have suggested alternative causal pathways: Palmer (2020) suggests that ethnic diversity in factors such as blood coagulation or stroke risk could drive the observed racial disparities. Here, rather than difference in genes directly involved in immune system response to SARS-CoV-2, individuals from certain racial or ethnic groups have an increased genetic predisposition towards particular health conditions. This disproportionate prevalence of health conditions that are themselves risk factors for Covid-19 then explains disproportionate infection and fatality rates.

Genetic differences as a causal explanation for racial disparities in Covid-19 has, therefore, clearly had uptake in the scientific and medical expert community. Interestingly, this has largely not been reflected in mainstream media coverage. Rather, many articles and opinion pieces, published in national newspapers, scientific magazines, and opinion sections of journals such as the Lancet, have pre-emptively responded to potential genetic explanations, emphasising the role of economic and social factors, and racism in particular, in producing these disparities, and embracing a concept of race as socially constructed rather than constituted by genetic difference (Gravlee, 2020; Morgan, 2020; Saini, 2020; Tsai, 2020; Zakrzewski, 2020).

The backlash against the search for genetic explanations for racial disparities has to be placed within the wider context of race-based medicine and the biological race concept more generally. Supposed biological racial difference has historically been used to justify social and medical inequalities (Gannett, 2001; Graves & Rose, 2006; Perez-Rodriguez & de la Fuente, 2017; Shaban, 2014). In the context of Covid-19, critics have worried about the reinforcement of narratives that naturalise inequality, as well as directing attention away from other explanations. In their open letter to the NIH, Yudell et al. (2020) worry that this genetic research could lead researchers and the public to “mistakenly point to innate racial differences instead of long-standing institutionalized racism and other underlying social, structural, and environmental determinants”. Yearby (2021, p. 76) argues that genetic research that tracks social race and ethnicity “reinforces racial inferiority”, and marks racial and ethnic minority groups as genetically abnormal, rather than addressing the real driver of racial inequalities: unequal power.

It is apparent that, whilst some researchers have pursued genetic explanations for racial disparities in the impact of Covid-19, this has been rejected by many researchers and journalists, and genetic claims have been subjected to intense scrutiny. These criticisms have involved rejecting race as a genetic category, but not the usefulness of the category in itself: there have been calls for increased collection of data broken down by race and ethnicity data (American Medical Association et al., 2021; McKenzie, 2021). Racial or ethnic groups have been embraced as centrally important categories through which to understand Covid-19 related differences, although the basis for these categories has been controversial.

### Sociostructural Narratives of Racial and Ethnic Disparities

If genetic explanations have been controversial, what is the alternative explanation for the observed disparities? Focus has been turned on the ways in which many social factors impact racial groups differently. Social, or sociostructural, explanations for the observed racial disparities have been proposed at various levels: these include differences in how people of different races are treated by the healthcare system once they contract Covid-19, differences in socioeconomic status (including occupation, amongst other factors) that result in differential exposure to coronavirus, as well as differences in environmental pollution and other factors that could affect pre-existing health status and the distribution of conditions such as cardiovascular disease and diabetes that are risk factors for the severity of Covid-19.

Garcia et al (2021) identify three mechanisms through which structural racism could be driving racial or ethnic disparities in the burden of Covid-19 in the US: healthcare access and quality, risk of exposure and weathering processes. There are well-documented racial disparities in both access to healthcare and quality of care (e.g., Manuel, 2018; Nelson, 2002). Ruprecht et al (2021), in a study carried out in Chicago, found that white participants had greater access to medical services and were more likely to have a medical provider they could contact if they needed Covid-19 testing, while Black and Latino participants were more likely to report barriers to accessing medical services. Azar et al. (2020) found that, in California, Black Americans were significantly more likely to be admitted to the hospital than white Americans, indicating that Black Americans may have more severe Covid-19 at the time of seeking medical care. They suggest this may be explained at least in part by barriers to timely access to healthcare.

There is also evidence for racial or ethnic differences in exposure to coronavirus. Residential and occupational segregation could put Black and Latino people at greater risk. Garcia et al. (2021) point to the possibility that racial minorities may have restricted capacity to physically distance due to institutional discrimination leading to high housing density. Additionally, racial minorities (in both the US and the UK) are disproportionately likely to be ‘essential workers’, who have been required to perform in-person work, increasing their risk of exposure (Figueroa et al., 2021; Office for National Statistics, December, 2020b; U.S. Bureau of Labor Statistics, 2019). Differential rates of incarceration could also contribute to different levels of exposure: US prisons, due to issues such as overcrowding, have seen multiple Covid-19 outbreaks (Simpson & Butler, 2020).

Another mechanism by which these differences might emerge is from pre-existing health disparities. Individuals from racial minority groups are more likely to suffer from cardiovascular disease or diabetes (McWilliams et al., 2009). This uneven distribution of comorbidities could explain the racial disparities in the burden of Covid-19, given that these conditions have been implicated as resulting in worse Covid-19 outcomes (Bhala et al., 2020). This could be utilised as part of a genetic narrative, if the disparity in comorbidities is taken to be resulting primarily from genetic differences (as with Palmer, 2020). However, this assumption has been criticized (Roberts, 2011, 2012). Alternatively, this could be part of a sociostructural explanation if the proximate cause for the differences in the burden of Covid-19 (the disparity in comorbidities) itself is taken to be the result of structural racism and its generational legacies of deprivation (Mendenhall & Gravlee, 2021).

Focusing on sociostructural reasons for racial health disparities in the context of Covid-19 shifts the focus from intrinsic characteristics of racial groups to the impacts of structural racial discrimination. In these explanations, racial categories are still taken to be important analytical categories, although these categories are not defined genetically but by social racial hierarchies. Social racial hierarchies explain the differences in housing, occupation, healthcare access and quality, and other factors that contribute to the disproportionate effects of Covid-19 on racial minority groups, through processes of both interpersonal and structural or institutional discrimination. This provides very different avenues for remedying these disparities. Ovsyannikova (2020) call for more candidate gene, epigenetic, and GWAS studies “across different racial and ethnic populations in order to identify genes and haplotypes associated with differential factors of infection and clinical outcome, as well as vaccine response” (214). In contrast, seeing these disparities as arising through sociostructural processes brings to the forefront social or political interventions, that might range from expanding access to healthcare, poverty alleviation programs, and community-based alternatives to prison.

Sociostructural narratives have achieved significant uptake, primarily in terms of widespread media coverage, but also within the epidemiological and public health literature outlined above. Although this is in part a continuation of longstanding debates around biological versus social explanations of racial difference, these narratives also acquired more urgency and force given the broader political context. In the USA (as well as in other areas, such as the UK), the crisis around police brutality and violence, and the responses of the Black Lives Matter protests, made structural racism a highly visible issue: in media coverage, the increased burden of Covid-19 and risk of police violence for Black people were often brought together (Paz, 2021; Resnick, 2020).

### Biosocial Narratives of Racial and Ethnic Disparities

Genetic and sociostructural explanations have therefore been placed in direct opposition to each other in the context of trying to grapple with the increasingly visible racial and ethnic disparities that have emerged during the course of the pandemic. Moreover, these two broad classes of explanation seem to lead towards different types of intervention. However, biological and social processes do not necessarily have to be understood as mutually exclusive explanations or in tension with one another: developing research is exploring the ways in which the biological and social become intertwined in the context of race.

Epigenetics, changes in DNA methylation that can be triggered by environmental change, provides a mechanism by which the social environment, including features such as psychosocial stress, discrimination and environmental pollution can reach into the body and enact physiological, and sometimes intergenerational, effects. It has therefore been suggested that racial health disparities can be explained through the effect of social racial hierarchies on biological processes (Kuzawa & Sweet, 2009; Sullivan, 2013). Gravlee (2020) emphasises that “environments, experiences and exposures have profound impacts on how our bodies develop”. He argues that systemic racism is “as much a part of biology as genomes are”, and systematic racial differences in factors such as access to healthy food, exposure to environmental pollution, the threat of police violence, or the stress of discrimination, influence a range of health conditions, including the severity of Covid-19.

The implications of this view of health disparities are complex. On the one hand, this could pave the way for a more nuanced understanding of the ways in which the social and biological interact; understanding the biological impact of social racial hierarchies could provide further motivation to address structural racism, and contribute to the urgency for implementing widescale policy solutions. Under this view, the focus shifts from race as a biological category to *racism* as a process which has biological effects (Kalewold, 2020). However, whilst epigenetics could shift our focus from innate difference to the biological effects of racism, there is also the danger of this research propelling narratives of “acquired inferiority of specific populations” (Meloni, 2017, p. 402). The implications of this biosocial perspective are ambiguous: understanding the impact of social racial hierarchies on biological or physiological processes could provide further motivation to address structural racism, although it could also divert attention away from broad social interventions and ‘biomedicalise’ racial disparities.

Although research in the area of the epigenetic effects of social or racial inequality is growing rapidly (Meloni, 2017), ‘biosocial’ explanations in the context of racial disparities in the burden of Covid-19 in particular have as yet not received scientific attention or widespread media coverage.

### General Lessons of Racial and Ethnic Narratives Around Covid-19

As has been shown, genetic, sociostructural, and biosocial narratives have all emerged in the context of trying to explain racial and ethnic disparities in the burden of Covid-19. Although some researchers have proposed genetic explanations, there has been forceful pushback against these narratives. Researchers and journalists have pointed to the capacity for social and structural factors to explain the observed disparities, as well as the kinds of policy interventions that these explanations license. This has led to broader conversations around structural racial injustice, with the hope that the stark disparities made visible during the pandemic could be a catalyst for wider societal change (Yancy, 2020).

The usefulness of racial or ethnic categories, at least in the context of understanding racial disparities in the US and UK, has not been in question during the pandemic. However, in broader discourse around the use of racial categories in medicine and public health, researchers have raised the question of when broad statements about racial groups and elevated or lowered risk of particular diseases can be justified. Valles (2012) examines claims that Black Americans are at higher risk of hypertension, and white Americans are at higher risk of cystic fibrosis, pointing to the heterogeneity of risk in subpopulations within these groups. Foreign-born Black immigrants do not have elevated risk of hypertension, and people with Finnish ancestry have very low rates of cystic fibrosis. Valles argues that we should increase the level of specificity in our characterisation of at-risk populations, otherwise we risk both imprecise or inappropriate targeting of these low-risk subpopulations and reinforcing naive biological race essentialism. In the context of Covid-19, UK data has shown that, although all South Asian people have an increased risk of death from Covid-19 compared to white people, those of Bangladeshi and Pakistani ethnicity are at significantly increased risk compared to those of Indian ethnicity, even after adjusting for factors such as level of area deprivation, household composition, socioeconomic status, education, and health or disability (Office for National Statistics, May 2020a). However, media reporting on these disparities has often not highlighted these differences, instead reporting on the increased burden of Covid-19 on South Asians as a group (e.g., Boseley, 2021). There are therefore similar questions to those raised by Valles around the correct level of specificity we should use when understanding Covid-19 disparities: when is it useful to talk about racial groups rather than ethnic groups, or more specific subpopulations? To what extent do we tolerate heterogeneity within at-risk populations for medical or public health purposes?

Another key aspect of understanding racial and ethnic narratives during the pandemic is the kinds of social responses or political interventions they engender. In general, both expert and public conversations around racial or ethnic disparities in Covid-19 have, to a significant extent, resisted individualising harms and resisted either placing blame on racial minority communities or casting these harms as natural and unavoidable. In the following section, I will turn to narratives that have arisen around ‘obesity’ or fatness in the context of Covid-19. I will show that the kinds of narratives and explanations that have emerged for the disproportionate impact of Covid-19 on fat people are very different: narratives of personal responsibility have been dominant, and even those narratives which point to structural factors still neglect structural harms to fat people as a social group. In Sect. 4 I discuss the possibility that these ways of understanding obesity are racialised, providing a ‘backdoor’ to individualising harms and apportioning blame to Black and brown people.

## Fatness and Health Disparities in the Context of Covid-19

Disparities in the incidence and severity of Covid-19 have been observed when comparing obese with lean individuals, with obesity typically defined as having a body mass index (BMI) of 30 or higher. A meta-analysis of 75 studies reported that obese individuals were more likely to contract Covid-19, were more likely to be hospitalised with Covid-19, and to die from Covid-19 (Popkin et al., 2020). Public Health England (2020) has reported similar trends, finding that the relationship between “excess weight” and Covid-19 risk persists even after adjustment for factors such as age, sex, socioeconomic status, ethnicity, and co-morbidities such as hypertension or diabetes.

The way in which these disparities have been examined and communicated during this pandemic, when compared to racial or ethnic disparities, is starkly different. The main narratives that emerged in the context of racial or ethnic disparities are either almost entirely absent, or are present in a different form, in the context of understanding the disproportionate burden of Covid-19 on fat people. In particular, sociostructural narratives that examine the structural impact of fat discrimination and fatphobia have been taken up by activists and some scholars, although these discourses have been marginalised within scientific or public understandings (see Sect. 3.3).

For some, the contrasting of these disparities might seem strange. We are increasingly exposed to arguments for race as a social category, whilst treating ‘obesity’ (or fatness) in this way is unfamiliar to many. However, there are reasons to think there are important similarities that allow for fruitful comparison. In both cases, there are disparities in employment, income, and access to quality healthcare, as well as interpersonal discrimination, cultural stereotypes, and stigmatising social norms (Brewis et al., 2011; Judge & Cable, 2011; Pausé, 2014; Saguy & Ward, 2011). These groups can be, and have been, conceptualised in various ways: either as biological categories, where biological difference is what separates groups from one another, and which paves the way for discourses that naturalise this difference and resist social intervention, or as social categories formed out of social processes that create hierarchies, providing avenues for rectifying this social injustice. There is a rich and growing body of literature that has investigated and advocated for a conception of fat people as a discriminated against social group (examples can be found in: Cooper, 2010; Hopkins, 2012; Mollow, 2015; Pausé & Taylor, 2021; Rothblum & Solovay, 2009; Saguy, 2012). Scholars have criticised the ways in which ‘obesity’ is defined in biomedical discourse, surrounding assumptions concerning the connection between body size and health, as well as how this biomedicalisation has served to justify discrimination and stigma and naturalise observed inequalities (Lupton, 2018 provides an overview; Eller, 2014 argues for the existence of *fat oppression*).

In terms of the academic and public discourse that existed pre-pandemic, there were similar strands of thought developing as in the case of racial or ethnic disparities in health: the investigation of the genetic basis of obesity is an ongoing research programme, as is the exploration of social or environmental dimensions to obesity. However, the ways in which certain narratives have been picked up, and the directions that research has pursued during the pandemic thus far has been markedly different. As racial and ethnic disparities in the burden of Covid-19 became clear, opening up conversations around structural racism and its effects on health, discourses around fatness and the disproportionate burden of Covid-19 have often been focused on personal responsibility, further stigmatising and marginalising fat individuals.

Before exploring these narratives, it is important to clarify terminology. The scientific and public health literature typically uses the term ‘obesity’, referring to individuals whose BMI is 30 or higher. However, the use of this term is controversial: scholars have argued that ‘obesity’ has normative and pathologising connotations (Wann, 2009). Lupton (2018, p. 3) argues that “to describe someone as ‘obese’ immediately places that person within the purview of medicine as someone who has the disease of ‘obesity’ and is therefore considered abnormal, inevitably healthy or at high risk of disease”. Therefore, fat activists and fat studies scholars typically prefer the term ‘fat’, rejecting both pejorative connotations of the word, as well as the biomedicalisation of larger bodies. Here, I will use the term ‘obesity’ in the context of discussing scientific research and media uptake of this research which relies on the term, and ‘fat’ outside of this context.

### Personal Responsibility and the ‘Second Pandemic’

Studies have found relationships between obesity (measured by BMI) and risk of infection, hospitalisation, or death from Covid-19 (Deng et al., 2020; Lighter et al., 2020; Popkin et al., 2020), although some have found associations only within some groups (e.g., for men but not women, as in Tartof et al., 2020). Within scientific literature and expert commentaries, obesity has been characterised as a “critical risk factor” (Kwok et al., 2020), a “big problem” (Kass, 2020), and an “Achilles heel” for Covid-19 (Muscogiuri et al., 2020). These scientific studies have been extensively reported by mainstream media outlets (e.g., Rabin, 2020) which have disseminated the message of obesity as a central issue in addressing the pandemic. Qanta Ahmed (2020), for The Daily Beast, argues that “getting into shape has become a matter of urgent national public health security”.

This scientific and public focus on obesity as a risk factor for Covid-19 has come along with shaming and blaming narratives that hold individuals accountable for their body size. In February 2021, the former television presenter Anthea Turner shared on Twitter a cartoon that had been circulating on social media (Davis, 2021). This cartoon depicted a fat woman in a wheelchair or mobility scooter, wearing a mask and holding a bag with the McDonald’s logo on it, telling a thin woman without a mask: “Put a mask on! You’re putting my health at risk”. Here it is presented as absurd for a fat person to blame a thin person for not adhering to mask guidelines: the real problem is not lack of mask-wearing, it is fatness in itself. The obituaries of fat people who have died from Covid-19 have been targeted by commenters who blame the death on the individual’s choice to be fat (Russo, 2020), and public figures have advocated shaming fat people and holding them responsible for their body size (Flint, 2020).

Aside from the bubbling up of fatphobia on social media platforms, government initiatives and policy proposals have also been put forward to address what has become characterised as a key issue during the pandemic. The UK Prime Minister, Boris Johnson, announced an ‘obesity crackdown’, with plans to introduce ‘anti-obesity measures’ including changes to labelling of particular foods, mandatory calorie information for restaurant food, and the ability for GPs to hand out ‘cycling prescriptions’ (Foster, 2020). Simon Stevens, NHS England’s chief executive, has called for individuals to adopt changes in diet and exercise (Helm & Campbell, 2020). The Centers for Disease Control and Prevention (CDC, 2021a, 2021b) stress that the “epidemic of obesity is impacting the severity of the COVID-19 pandemic”, and list individual measures aimed to induce weight loss. These proposed measures target the individual, either through urging individual behaviour change directly or through changing incentive structures to prompt individual behaviour change.

Another way in which obesity has repeatedly been framed is in terms of the ‘second pandemic’ (Chua & Zheng, 2020; Lockhart & O’Rahilly, 2020; Stefan et al., 2021). Changing population trends in body size have long been termed the ‘obesity epidemic’, with usage of the term dating back at least to the late 1990s (Mokdad et al., 1999). This term has been criticised on both scientific grounds and in terms of how the epidemic framing stigmatises and imbues the ‘problem’ of fat bodies with emotional urgency (Saguy & Riley, 2005). In the context of the Covid-19, this language appears to have been ramped up. Stefan et al (2021), in a paper titled *Global pandemics interconnected—obesity, impaired metabolic health and COVID-19*, focus on obesity as an ‘accelerator’ of severe Covid-19 and suggest that bariatric (weight loss) surgery could be an “important therapeutic option”. Both the British Obesity and Metabolic Surgery Society and the American Society for Metabolic and Bariatric Surgery have urged their respective governments to increase access to bariatric surgery, relying on the link between obesity and increased risk of Covid-19 (Lay, 2020; Schaffer, 2020). The calls for bariatric surgery are framed as an emergency measure to stem the spread of the ‘second pandemic’, necessary to adequately deal with the effects of the first. Again, this measure is focused on individual, with correlations between obesity and Covid-19 legitimising invasive individual medical intervention for the public good.

Overall, in the scientific literature, public health pronouncements, and wider discourse, narratives around personal responsibility and the stigmatization of fat bodies have had significant uptake (Monaghan, 2021). However, there has also been resistance to these narratives in varying degrees: some have attempted to move away from personal responsibility and blame while maintaining that obesity is an inherently risky biological category, whilst others have taken up structural fatphobia as a cause of disparities in the burden of Covid-19.

### Social and Environmental Narratives

Some journalists and experts have pointed to the role of social factors such as poverty, inequity, and stigma as drivers of obesity and the observed health disparities (Senthilingam, 2020; Wu, 2020). Stein and Ometa (2020) highlight the role of socioeconomic status, food insecurity, restricted access to a healthy diet and recreational facilities, distance to stores, and exposure to ‘obesogenic environments’ as drivers in disparities in rates of obesity in particular populations. Storz (2020) points to the ‘unprecedented obesogenic environment’ that has been created during the pandemic, due to changes in sedentariness, diet, and psychological stressors, and its potential impact on childhood obesity.

This understanding of obesity tries to shift the focus from individual behaviours to broader features of the social and material environment that individuals find themselves in. This approach builds on broader research on the ‘obesogenic environment’, defined as “the sum of influences that the surroundings, opportunities, or conditions of life have on promoting obesity in individuals or populations” (Swinburn et al., 1999). These influences could include diverse features such as urban design, terrain, climate, food insecurity, income, education, neighbourhood crime, and social support (Kirk et al., 2010).

These approaches try to avoid blaming or shaming fat people as individuals, instead looking to broader ‘ecological’ drivers of trends in body size. However, research into the role of ‘obesogenic environments’ typically continues to maintain that obesity is a medical or biological category that is inherently diseased. Whilst these approaches push back against models that attribute responsibility to individuals, they nevertheless carry the potential to stigmatise fatness. Colls and Evans (2014) point to the fundamental differences between accounts that depend on the assumption that fat bodies are inherently pathological and those which offer alternative accounts of fatness that deny the legitimacy of this assumption. They suggest shifting the emphasis away from identifying environmental factors which make a body fat (and necessarily problematic) towards how particular environments make living as a fat person problematic.

Therefore, somewhat analogously to the case of racial or ethnic disparities, we see a pushback against individualising models that can drive stigma, and towards an acknowledgement of the role of social and environmental factors (Pearl, 2020). This builds upon the growing awareness within medicine and public health around weight stigma (Rubino et al., 2020).^3^ However, in contrast to the sociostructural narratives around racial or ethnic disparities, fatness is still positioned as in itself inherently unhealthy or diseased: it is still a thoroughly biological, rather than social, category.

### Fatphobia and Structural Discrimination as a Driver of Health Disparities

The individualising model has been a dominant component of discourses around the disproportionate burden of Covid-19 on fat people, alongside some pushback against blaming narratives, which still shares the conception of fatness as a biological category of disease. However, some voices have rejected this characterisation, in whole or in part, emphasising the ways in which worse health outcomes can stem from social discrimination, marginalisation and stigma.

Townsend et al. (2020) urge more consideration of the role of interpersonal bias and discrimination in understanding the relationship between obesity and Covid-19. They emphasise that the problem of weight stigma has been neglected during the pandemic, stressing that weight bias exerts a powerful effect in both medical and public settings, and is still prevalent amongst healthcare providers. This weight stigma could, for example, lead to fat people being less likely to seek medical care in a timely fashion, and more likely to receive a poorer standard of care, resulting in worse outcomes. The authors acknowledge that biological susceptibility to increased mortality is likely, although healthcare avoidance could act synergistically with this susceptibility. Whilst these authors emphasise the role of weight stigma in driving health disparities, they leave medical conceptions of obesity largely intact.

Pausé et al. (2021a) tackle squarely the problematisation of fatness during the Covid-19 pandemic. They trace the ways in which fat bodies have been constructed as a major health problem and a burden on the health system. This construction relies on the questionable assumptions that fatness is central to an individual’s health status, that it is mostly under the control of individual choices, that weight loss is a realistic and achievable goal, and that BMI is a good predictor of current and future health status. Governments, public officials, researchers, and media outlets have seized upon obesity as a risk factor for Covid-19, even when the evidence has been ambiguous, using this to push for obesity prevention measures, weight loss strategies, and proposals to ‘ration’ healthcare away from fat people if necessary. They propose moving away from ‘problematising’ fatness towards a vision of radical health justice. Pausé and colleagues emphasise that the weight-centred paradigm has not reduced fatness or improved the health of fat people, as well as highlighting the potential for fat stigma and oppression to increase the vulnerability of fat people to negative impacts of the pandemic.

This approach shifts the understanding of fatness as a biological or medical category to a social category. What fat people as a group share is not one particular metabolic state or one kind of health status, but rather a position on the social hierarchy. The social positioning of fat people in this way means that they experience discrimination, stigma, reduced quality of and access to health care, reduced income and education opportunities, and so on, due to the social meanings attached to body size. As with the racial or ethnic disparities, understanding fatness as a social category opens up the possibility and urgency of interventions that address structural *fat discrimination* and the ways this discrimination has been amplified during the pandemic, rather than fatness as a problem in itself. This might mean interventions that tackle medical fatphobia, employment discrimination, or poverty.

Although some social sciences and humanities scholarship has emerged that attends to structural fat discrimination in the context of Covid-19 (Bessey & Brady, 2021; McPhail & Orsini, 2021; Monaghan, 2021; Pausé et al., 2021a, 2021b), overall, this narrative of structural fat discrimination has received far less attention than those which hold onto fatness as a biological category, whether that be coupled with personal responsibility and blame or highlighting the negative effects of weight stigma.

### General Lessons of Narratives of Fatness and Covid-19

In contrast to narratives around racial and ethnic disparities, genetic narratives have been conspicuously absent in the case of fat-thin disparities, despite an ongoing research programme into the genetics of obesity (Albuquerque et al., 2015; Bell et al., 2005). Media reporting has mentioned genetics in passing as one factor that drives differences in body size, although there has not been widespread attention to genetic narratives for fat-thin health disparities during the pandemic. Zhu et al. (2020) carried out a study which found a significant positive relationship between individual genetic predisposition for an obese BMI and the risk of severe Covid-19, indicating a role for obesity-related genetics. However, in comparison to the scientific research on the genetic basis of racial or ethnic disparities in the burden of Covid-19, research into obesity-related genetics and Covid-19 has been scarce.

One potential explanation for this is the different ways that genetic narratives are deployed and the divergent landscapes of responsibility and intervention they open up in different contexts. Genetic concepts of race have been used to naturalise inequalities and close off possibilities for social change. In contrast, genetic understandings of obesity often serve the function of escaping narratives of personal responsibility or blame, by putting forward a picture of obesity as a condition outside of individual control. Although any clear link between this absence and particular social or political agendas cannot be definitively discerned, an emphasis on the role of obesity-related genetics and poorer Covid-19 outcomes amongst fat people would conflict with the characterisation of obesity as either a matter of individual responsibility and bad choice, or in terms of a public health emergency we must take drastic, often individual-targeted, measures to abate. In the context of the pandemic, where individualising and shaming narratives have been amplified (perhaps, as Pausé et al., (2021a, 2021b) suggest, as part of a deflection from governmental and structural failures), research or general speculation into genetic components of obesity and therefore the observed health disparities may have seemed unattractive.

Additionally, narratives that shift focus from individual behaviours to social or environmental factors have often not involved a shift in the understanding of the category of fatness itself. This is in contrast to the pushback to genetic concepts of race from scholars and journalists who have emphasised race as a social, not biological, category. The call to refrain from thinking about racial or ethnic health disparities during the pandemic as arising from innate biological difference or inferiority, and arguments for focusing on the effects of racial discrimination that flow from socially constructed racial hierarchies, has received sustained attention in academic literature and media reporting. In contrast, the work of scholars and activists who have emphasised the effects of fat discrimination have been relatively marginalized in terms of uptake in both scientific research or practice, media coverage, and public discourse.

This may be due to the entrenchment of core assumptions of obesity science and the prevalence of fatphobia across many societies (Bacon et al., 2001; Bombak, 2014; Robinson et al., 1993). It is beyond the scope of this paper to give an adequate evaluation of the extent to which obesity is or is not a meaningful scientific or medical category (Lupton, 2018 provides one perspective on these ongoing debates). However, it is important to note that shifting the focus to fatness as a social category need not be a rejection of biomedical knowledge in its totality. Firstly, a rejection of particular BMIs or body sizes as intrinsically unhealthy does not entail the claim that all fat people are healthy or that there are no distinctions to be made between subpopulations. Metabolic science is increasingly complicating the relationship between BMI and metabolic dysfunction (Mathew et al., 2016). To return to the argument about specificity from Valles (2012), we could argue that targeting fat people, when there is evidence of heterogeneity of risk within this subpopulation, has significant undesirable consequences. This could legitimise focusing on more fine-grained distinctions, such as measures of metabolic health. Secondly, as with biosocial understandings of race, we would expect structural fat discrimination and weight stigma to affect physiological processes and contribute to worse health outcomes. A fuller understanding of the dynamics of fatphobia and structural fat discrimination will likely include investigation of the biological consequences of this discrimination (Warin, 2015 calls for this engagement with ‘relational biology’ in fat studies).

## Connections Between Racism and Fatphobia in the Context of Covid-19

Thus far I have discussed race or ethnicity and fatness in isolation from one another. Although the narratives that have arisen around them have often been disconnected and developed in diverging ways, this is ultimately an artificial separation. US data indicates that there is a higher prevalence of obesity within Black and Hispanic groups compared to white people (CDC, 2021a, 2021b). UK data suggests that there is a higher prevalence of obesity amongst Black adults compared to white adults, and a higher prevalence of obesity amongst Black and Asian children compared to white children (Public Health England, 2019; Sport England, 2020). We would therefore expect that anti-fat discrimination, weight stigma, and their attendant social and biological consequences, will disproportionately fall on racial minorities.

Beyond the statistical data, there is emerging research which explores the ways in which the fat body is racialised, and the connection between anti-fatness and anti-blackness. Sabrina Strings, in her 2019 book *Fearing the Black Body: The Racial Origins of Fat Phobia*, tracks the ways in which anti-fat bias has historically stemmed from racialised conceptions of the ideal body. As race science grew in the seventeenth and eighteenth centuries, the demarcation of racial differences in beauty became one part of the racial schemas that were being developed. Fatness grew increasingly linked with stupidity, greediness and laziness, as well as with Blackness. Thinness, in turn, became linked to whiteness. In nineteenth Century America, thinness became folded into a specifically Anglo-Saxon white identity, and held up as an “American beauty ideal”; fatness was considered a “racially inherited” property of groups positioned as inferior, that included the Polish, Irish, and Italians (Strings, 2019, p. 159). Strings brings the story of anti-fat bias up to the present day, noting how studies which emphasise the dangers of obesity, and particularly those that focus on Black women, receive widespread press attention, while conflicting findings are often ignored or downplayed by the media (ibid, p. 207). This history illustrates the ways in which the fear of the Black body has propelled fatphobia and the construction of the thin ideal.

Harrison (2021) and Shackelford (2021) and both see not only a connection, but an intimate relation between Blackness and fatness. Shackelford asks: “What if instead of believing that Blackness and fatness are separate embodiments that we connect the shrinking and active killing of Blackness as the same violence that asks us to shrink and disappear all fat bodies?” (2021, p. 257). For Shackelford, “the spectrum of punishment and violence for fat people” is “always in direct relationship with anti-Blackness”. Harrison argues that “it is through the unremarkability and unpleasantness of how fatness dressed Black flesh that created the structures that necessitate the marginalization of both identities” (2021, p. 35). Both Shackelford and Harrison connect racist police violence with anti-fatness, pointing to the case of Eric Garner and the police union lawyer who claimed Garner died from “being morbidly obese” (Shackelford, 2021, p. 257). This is argument for seeing anti-Blackness and anti-fatness as both historically co-constituted (“from the moment white Europeans saw fat Africans, the science that followed was intended to always separate them from the rest” (Harrison, 2021, p. 98)) and conceptually entangled: we cannot resist one without resisting the other.

An appreciation of this entanglement is necessary to make sense of the consequences of the narratives that have arisen around the disparities observed during the Covid-19 pandemic. It is the case that there has been sustained, and positive, attention directed towards the effects of structural racism as a defining feature of the pandemic. However, the same cannot be said in the case of fatness: whilst some scholars have carried out important work to focus on the heightened effects of structural fatphobia during the global crisis, the pandemic has largely led to ramping up of stigmatizing attitudes and narratives of personal responsibility and blame. Crucially, given the ways in which race and fatness, and racial and anti-fat discrimination, have been intertwined, historically, empirically, and conceptually, the lack of attention to structural fat discrimination in favour of an individualising and blame-centred approach provides a way for these tendencies to *nevertheless* disproportionately affect racial minorities, fuel racial hierarchies, and perpetuate white supremacy.

## Conclusion

I have shown that the narratives around the disproportionate burden of Covid-19 on racial or ethnic minorities, and those around the burden on fat people, have diverged in important ways, which indicate particular conceptions of the kinds of categories we are dealing with and license particular types of social or political interventions. In the case of racial disparities, genetic narratives achieved some uptake in the scientific literature, expert commentaries, and popular media, although there was significant resistance. Sociostructural narratives came to be the most prominent alternative to genetic narratives, fuelled perhaps in part by the broader social and political context of 2020 which made the issue of structural racism highly visible. The landscape of the debate was informed by an understanding of race as either a biological or a social category. Whilst some scholars proposed biosocial narratives, these have not received similar levels of attention. In the case of fat-thin disparities, in contrast, the most prominent narrative was that of personal responsibility, which was often accompanied by stigmatizing, blaming or shaming. Here, fatness (or ‘obesity’) is positioned as inherently risky, or a disease in itself (that is, a thoroughly biological category, where biological difference is the central explanatory factor for the observed disparities). The most prominent alternative narrative was one which emphasized social and environmental factors in an effort to combat weight stigma or the individualizing of blame, but nevertheless also accepted fatness as a biological category. Narratives which identify structural fat discrimination as a driver of Covid-19 disparities, and conceptualise fatness as a social category, have been present, although not prominent. Notably, explanations that pick out structural fat discrimination have been far less prevalent both in the scientific literature and in media coverage than those which highlight structural racism. I have suggested, given scholarship that indicates deep connections between race and fatness, that the lack of attention to structural fat discrimination has racialized effects. Therefore, it is important to consider racial and fat-thin disparities in tandem. More attention must be given to how we understand and discuss the social disparities that have characterised the development of the pandemic, and how intersecting and interconnected hierarchies have given rise to these inequalities.

## Footnotes

1 I use ‘fat’ here, as the preferred term amongst fat activists and fat studies scholars, who reject the stigmatising and pathologising connotations of the term ‘obesity’ (Wann, 2009). In Sect. 3, I will use ‘obesity’ when referring to scientific research that relies on this term, and ‘fat’ outside of this context.

2 By ‘narrative’, I mean an explanatory story about the emergence and persistence of these disparities as represented in scientific literature and public discourse. This story structures discourse in particular ways: as a narrative builds, further discourse either buys into the given narrative or positions itself against it. Explanation is a central component of these narratives (i.e., they involve the adoption of a particular causal story that provides an explanation of observed disparities). However, I use the term ‘narrative’ here, rather than explanation, in order to capture not only the explanatory structure, but also the ways in which a story becomes built up. This story involves, more broadly, connections between proposed explanations of the disproportionate burden of Covid-19 on racial/ethnic minorities or fat people and ethical or political questions of responsibility and intervention. Furthermore, although within the narratives I identify, there may be specific differences in the causes picked out as responsible (such as identifying one gene or set of gene expression patterns over another) and therefore in some sense different explanations, the thematic similarity between these explanations leads to similar ethical or political implications, as well as similar uptake and responses to these explanations in the public arena.

3 In 2020, a joint consensus statement was produced by a panel of 36 experts, which pledged to eliminate weight bias and ‘stigma of obesity’ (Rubino et al., 2020). This statement draws on a wealth of empirical evidence relating the harmfulness of weight stigma. However, it is clear that in this statement fatness is conceived as a strictly biological category: the authors advocate seeing obesity as a chronic disease and recommend metabolic surgery as a potential treatment pathway.
